# Personalised care for people with excessive alcohol use following an episode of self-harm: a mixed methods community case study in a psychiatric liaison team

**DOI:** 10.3389/fpsyt.2025.1608804

**Published:** 2025-08-22

**Authors:** Sarah Wigham, Elizabeth Titchener, Katherine Jackson, Eileen Kaner, Eilish Gilvarry, Amy O’Donnell

**Affiliations:** ^1^ Population Health Sciences Institute, Newcastle University, Newcastle upon Tyne, United Kingdom; ^2^ Cumbria, Northumberland, Tyne and Wear NHS Foundation Trust, Newcastle upon Tyne, United Kingdom

**Keywords:** alcohol, non-dependent, rapid access, intervention, self-harm

## Abstract

**Background:**

Excessive alcohol use is common among people presenting to emergency departments with self-harm; however, this group face barriers accessing appropriate support. This study aimed to evaluate a rapid access personalised face-to-face service developed to address this gap and explore wider implementation opportunities.

**Methods:**

We conducted a service evaluation with a mixed methods convergent design. An NHS data custodian extracted and anonymised electronic health records data prior to sharing with the research team for analysis using descriptive statistics and non-parametric tests. Qualitative semi-structured interviews were conducted with patients and clinicians and analysed thematically. Quantitative and qualitative data were integrated, and meta-inferences drawn.

**Results:**

Patients accessing the service (n=68) were mostly female (61.8%), white (83.9%), mean age 35 years (range 19-69), and most experienced additional mental health conditions alongside excessive alcohol use. Preliminary exploratory calculations comparing baseline to follow-up Recovery Quality of Life (ReQoL-20) scores suggested positive change. Three themes were identified from interviews with patients (n=11) and clinicians (n=7): (1) what the service added: rapidly plugging a recognised care gap for people using alcohol excessively but who are non-dependent and have poor mental health (2) what worked well: tailored relational support that builds recovery positive social networks and personal coping strategies (3) what could be improved: opportunities/challenges to sustaining and scaling-up the service.

**Conclusions:**

The findings contribute to an evidence gap in appropriate care for patients with excessive alcohol use, self-harm and poor mental health. Whilst limited to one service, the findings highlight what patients valued, opportunities for implementation in other contexts, and thus have relevance internationally.

## 1 Introduction

Evidence suggests excessive alcohol consumption (defined in the UK as >14 units a week: a unit equating to eight grams of pure alcohol) (1), and mental health conditions commonly co-occur (2–6). Around a third of people who meet diagnostic criteria for major depressive disorder drink alcohol excessively (6–8). Experiencing excessive drinking and mental ill-health concurrently is associated with increased self-harm risks (9–13). Excessive drinking is highly prevalent amongst emergency department attenders in the UK and internationally (13, 14). A UK study showed alcohol involvement in 58.4% (n=11,556) of emergency department attenders with self-harm (14). UK prevalence of non-suicidal self-harm is 6.4% of the general adult population (15). Clinical guidelines define self-harm as intentional self-poisoning or self-injury regardless of the purpose (suicidal or non-suicidal) (16).

Research suggests treating excessive alcohol use and mental ill-health in parallel is important to ensure positive outcomes (4, 17). Further people with excessive alcohol use and mental ill-health frequently have multiple and complex needs, meaning that holistic approaches, that are coordinated across different health and care service providers and are responsive to local contexts are required (18–21). Evidence shows that people with excessive alcohol use and self-harm often face barriers to appropriate support, including gaps in service provision and limited understanding by services of their wider socio-economic circumstances (17, 22–25). Preventative approaches are also required for those who may not actively seek support for excessive drinking or who do not meet eligibility criteria for specialist addictions services (26–28).

### 1.1 Context

Research suggests alcohol brief interventions in the emergency department are beneficial and cost effective (29–31) however less research has specifically explored the use of brief interventions for people with alcohol misuse and self-harm and the findings from these studies are mixed. Previous research showed that a brief intervention for alcohol misuse following self-harm did not significantly reduce alcohol consumption or subsequent self-harm (32). The intervention was a 30-minutes interview with an alcohol specialist nurse and the authors suggested that longer interventions may be required. A systematic review of psychological interventions to reduce alcohol use found they led to reduced self-harm but had no impact on suicidal ideation (20). A Delphi consultation was conducted to identify core components of a brief intervention for emergency department attendees with self-harm and substance use difficulties; the authors planned feasibility testing of the intervention which comprised weekly phone calls for one month (33).

To address the care gap faced by people with excessive alcohol use who self-harm, a rapid access service (RAS) was developed and provided by the Psychiatric Liaison Service of a large NHS trust in North East England. This region has high levels of alcohol-related morbidity and mortality (34), above average deprivation (35), high numbers of people seeking mental health support (36) and high suicide rates compared to other parts of England [14.5 suicides per 100,000 people in the North East compared to 7.3 in London (37)]. The RAS clinic provided rapid access to short-term personalised face-to-face care for people identified as drinking excessively and accessing emergency services after a self-harm episode.

The RAS service was delivered at a community venue by a specialist substance use nurse with mental health experience. Patients presenting in emergency care at a local hospital following an episode of self-harm, were reviewed by RAS clinicians and those with excessive but not dependent level alcohol use (i.e. who psychiatric liaison clinicians did not consider suitable for or who did not meet criteria for referral to specialist addictions services) were eligible for RAS rapid access support. Care typically consisted of the offer of three personalised face-to-face sessions, including psychoeducation, and motivational interviewing-based alcohol and mental health support. Sessions were held weekly, with patients offered the next available session following the self-harm episode, usually the following week. After three sessions, patients were referred or signposted to sources of longer-term care as needed e.g. counselling or support from third sector organisations. This study aimed to (1): understand the care journeys of patients accessing the RAS service; and (2) explore implementation experiences and identify opportunities for transfer to other settings.

## 2 Methods

This mixed methods service evaluation used a convergent approach collecting and analysing quantitative and qualitative data in two parallel parts. Part 1: collection and analysis of quantitative electronic health record data. Part 2: qualitative interviews with patients and clinicians. The quantitative and qualitative data were then integrated, to generate deeper understanding of how the RAS service supported people (38).

### 2.1 Part 1

Electronic health data were accessed via the UK NHS Clinical Record Interactive Search system (CRIS). The research team provided an NHS data custodian with instructions regarding specific data needs, and the custodian conducted searches, extracting relevant data on their behalf and ensuring anonymisation before sharing with the research team for analysis.  Data were extracted for all patients referred to the RAS from 23/2/23 (when the service started) to 31/8/24. The RAS ran one day a week and only when the specialist substance use nurse was available, so for some weeks during this period the service was not operational.

Extracted data included socio-demographic characteristics and postcode to calculate Indices of Multiple Deprivation (IMD), a measure of deprivation across seven domains (income, employment, education/skills/training, health/disability, crime, housing/services and living environment) (39). Data were also extracted on mental health or neurodevelopmental diagnoses, referrals to follow-up organisations, and Recovering Quality of Life (ReQoL-20) scores. The ReQoL-20 is a 20-item self-report measure of outcomes identified by respondents as important to recovering their quality of life (40). Reliable improvement is indicated by increases of ≥10 points while decreases of ≥10 points indicate reliable deterioration (40). Patients accessing the RAS were assessed with the ReQoL-20 at baseline (week 1) and intervention end (week 3).

Quantitative data including patient demographic and clinical characteristics, baseline and post-intervention ReQoL-20 scores and post-intervention service referrals were analysed with descriptive statistics and exploratory non-parametric tests. Pre/post-intervention ReQoL-20 scores were compared with Wilcoxon signed rank test using SPSS statistical software (41).

### 2.2 Part 2

Semi-structured interviews were conducted with patients accessing the RAS and clinicians involved in the design, commissioning, and delivery of the clinic.

#### 2.2.1 Recruitment

*Patients.* Inclusion criteria were patients >18 years of age with excessive alcohol use who had accessed the RAS after presenting to the emergency department with self-harm. Patients were screened for eligibility by a RAS clinician, provided with information about the service evaluation, and asked if they were interested in taking part and agreeable to their contact details being provided to the research team. Those expressing interest were then contacted by the research team. Patient participants were provided with support organisation contact details and a £20 voucher.

*Clinicians.* A range of clinicians directly and indirectly involved in the delivery, management, referral to and commissioning of the RAS clinic were recruited through purposive sampling. A RAS clinician facilitated contact between the researchers and potential clinician participants who were then contacted directly by the research team.

#### 2.2.2 Data collection

Semi-structured interview topic schedules were developed for patients and clinicians (see online **Supplementary Materials: OS1, 2**). Interviews with patients who had accessed the RAS explored their experiences including what worked well, and barriers/facilitators to their engagement. Patient demographic information was gathered using a structured form (OS3). Clinicians’ interviews covered the design and scope of the clinic, what worked well, challenges to delivery, and potential to transfer the service to other settings.

Interviews with all participants were conducted via MS Teams, audio-recorded and transcribed; transcripts were anonymised, and participants allocated a pseudonym. All participants provided informed consent before taking part in an interview.

#### 2.2.3 Analysis

Interview data were analysed using thematic analysis combining deductive and inductive approaches during four iterative phases: (i) data familiarization; (ii) defining a coding framework; (iii) identifying themes/subthemes; and (iv) refining themes/subthemes (42, 43). We defined a-priori themes based on the evaluation questions, then inductively derived codes and subthemes from the data, mapping these onto the a-priori themes. Patient and clinician interview data were analysed together with between-group similarities and differences explored. Two researchers completed data analysis, meeting regularly to discuss coding, with 20% of transcripts double-coded independently. NVivo software was used to support data management and analysis.

Key findings from both Part 1 and 2 were combined: descriptive statistics from the quantitative data and quotes from the qualitative data were integrated in tables, allowing comparison, discussion of convergence and divergence and drawing meta-inferences (38).

### 2.3 Approvals

A service evaluation application was approved by the NHS Trust Research and Development Department (23/5/24: SER-24-277). The local NHS CRIS database oversight committee reviewed the service evaluation protocol and approved collection of retrospective quantitative health record data.

## 3 Results

### 3.1 Exploratory quantitative analysis of health data

Health record data were gathered for 68 patients referred to the clinic from 23/2/23 to 31/8/24. The characteristics of those people attending one or more of the RAS sessions offered are shown in **Table 1**. Thirty-eight patients (55.9%) had one or more co-occurring mental health condition (**Table 1**). Being a new service, the team were consolidating service protocols, and ReQoL-20 scores were not always completed, and some participants did not attend all appointments, meaning follow-up scores were sometimes unavailable: ReQoL-20 scores at baseline and follow-up were available for fifteen people. Mean ReQoL-20 score at baseline was 30.8 (range 14-53) and 46.6 (range 23–69) at follow-up (final session), suggesting improvement across the cohort (40). The mean change in scores from baseline to follow-up was 15.8 (range 6 - 39). Of these 15 patients, four (26%) moved from the clinical (0-49) to non-clinical range (≥50). A Wilcoxon’s signed rank test showed improvements in median ReQoL-20 scores from baseline to follow-up (p<.001).

**Table 1:** Characteristics of patients referred to the clinic (n = 68).

Demographic and clinical characteristics				N		%
Sex						
Male				24		35.3
Female				42		61.8
MV				2		2.9
Ethnicity						
White				57		83.9
Mixed				1		1.5
MV				10		14.6
DNA				23		33.8
*Age:* mean 35.50 years (SD: 13.41); range 19–69						
*IMD*: median 3.41 (SD: 2.38); range 1-10						
Co-occurring mental health conditions						
Personality disorder				6		8.8
Eating disorder				1		1.5
Post-traumatic stress disorder (PTSD)				3		4.4
Autism				4		5.9
Anxiety				14		20.6
Depression				19		27.9
Attention Deficit Hyperactivity Disorder				4		5.9
Head Injury				3		4.4
Bipolar disorder				1		1.5
Obsessive-compulsive disorder				1		1.5
Psychosis				1		1.5
*Onward referrals (n):* Social prescribing (1); crisis team (1); veterans support (1); Relate relationship counselling (1); community treatment team (2); citizens advice (2); community recovery (7); third sector mental health support (5); neurodevelopmental assessment (2); talking therapies (6); bereavement counselling (1); cocaine/narcotics anonymous (2).						

Exploratory analysis of change in ReQoL-20 scores (n=15)						
Intervention phase	Mean	Range	Median			
Pre-intervention	30.8	14–53	26			
Post-intervention	46.6	23–69	45		p<.001	

DNA, did not attend; SD, standard deviation; IMD, index of multiple deprivation; MV, missing value.

### 3.2 Qualitative interviews

Eleven patients and seven clinicians accessing/involved in operation of the RAS participated in an interview (**Table 2**). Clinician participants included a specialist drug and alcohol nurse who delivered RAS sessions and other members of the psychiatric liaison multidisciplinary team, representing those with different levels of involvement in the RAS so facilitating different perspectives. For example, participants included a psychiatrist and a psychiatric nurse who while not involved in delivering RAS sessions, were involved in the clinical care of patients accessing the RAS and decisions on their referral to the RAS. Other clinicians interviewed included those who were involved in managing the operational aspects of the RAS clinic for example facilitating organisational processes required for setting up the new service within the pathway and developing service protocols. Data analysis identified three themes: (1) What the service added: rapidly plugging a recognised care gap for people using alcohol excessively but who are non-dependent, who self-harm and have poor mental health; (2) What worked well: tailored relational support that builds recovery positive social networks and personal coping strategies; and (3) What could be improved: opportunities and challenges to sustaining and scaling-up the RAS service. Quotes are annotated (P: patient or C: clinician).

**Table 2:** Characteristics of the qualitative interview participants.

Patients (n = 11)	n	%	Clinicians (n = 7)	n
Age			Team Manager	1
Mean	45		Nurse Consultant	1
Range	24–57		Specialist Nurse (Drug and Alcohol)	1
Gender			Consultant Liaison Psychiatrist	1
Male	7	64	Social Worker	1
Female	4	36	Psychiatric Nurse	1
Ethnicity			Clinical Manager	1
White	10	91		
Not known	1	9		
Index of multiple deprivation decile				
Mean	4			
Range	1–9			
Education				
School leaving qualifications	7	64		
Vocational qualification	1	9		
First degree	2	18		
Not known	1	9		
Marital Status				
Single	1	9		
Married or living with partner	4	36		
Divorced or separated	5	46		
Widowed	1	9		
Employment status				
Full-time paid employment	6	55		
Part-time paid employment	1	9		
Not in paid employment	3	27		
Not known	1	9		

#### 3.2.1 Theme 1. What the service added: rapidly plugging a recognised care gap for people using alcohol excessively but who are non-dependent and have poor mental health

Patients eligible for RAS support were drinking excessively, but not currently dependent on alcohol and had accessed accident and emergency with self-harm. By supporting this specific population, clinicians and patients highlighted the novel contribution to care provision:

‘Especially for people that fall down the gap, so don’t quite belong in the specialist drug and alcohol services. They don’t quite belong in mental health services. But they need help and it’s an unmet need’ (C2).

Participants indicated the community-based location differentiated the offer from existing clinical services, important for this group who may not feel comfortable attending specialist addictions services due to stigma or not feeling they required that level of support. One patient said: ‘*I didn’t particularly want to see people who were in worse positions*’ (P2). Another patient expanded on this:

‘I found … it was quite a quiet place; so I go to [addictions service] which is very clearly marked what it is … and I sometimes get anxious that when I’m going in I might be seen; everybody knows what sort of place it is.’ (P6).

A clinician highlighted the need to keep the clinic ‘*out of drug and alcohol services’* (C1):

‘…didn’t have signs up and it wasn’t so noticeable so [patients] were given anonymity around why they were there. [Addictions service] is in the middle of the city centre. Yes, it’s great for accessibility, but for other clients it’s not. It’s too busy. Too many people stand around outside. There’s the pub opposite.’ (C1).

Participants suggested the combined support for mental health and excessive alcohol use provided by RAS distinguished it from existing services:

‘A lot of people feel that they have mental health difficulties, and they won’t be touched, or they’ll be excluded, because of their alcohol use, and that isn’t the case here. We’ll carry out mental health interventions and substance misuse interventions, not one or the other, we do both for the individual’ (C3).

One patient described being admitted to hospital prior to accessing RAS but felt their full needs were not addressed:

‘The doctors treated the physical symptoms, which obviously was to stop the alcohol which I did successfully … I was sent home from hospital … but they hadn’t dealt with the mental side of things’ (P10)

Patients and clinicians valued the rapid support the service provided. One patient said: *‘it meant that I had access to … support very quickly’ (P10).* An emergency department clinician valued being able to offer timely appointments:

‘As a clinician, it’s an incredibly valuable resource to be able to offer someone something tangible in the ED [emergency department], because there’s so little to, often, offer people. Obviously, you’ve done your intervention in the ED. You’ve done your psychosocial assessment, which has, hopefully, left the person feeling safer to leave and with a safe plan in place. But there’s something incredibly validating to be able to give somebody an appointment there and then’ (C2).

#### 3.2.2 Theme 2. What worked well: tailored relational support that builds recovery, positive social networks and personal coping strategies

Patients described characteristics of the RAS provider (a specialist substance use nurse) that helped them open-up about their difficulties with alcohol, mental health, and reasons for drinking including being approachable, able to establish rapport, and having active listening skills:

‘The way he spoke to you; it was like talking to one of your mates. Like he didn’t think anything bad of you … when I was telling him about flying my drone and things like that, when I went in for my next session … he’d ask me how that was going’ (P8)

‘He just got it and understood that … what I was doing and why I was doing it and that was comforting because it was tough, at the time and he sort of helped me get to the bottom of a few things which maybe I wouldn’t have got there by myself, or it would have took me a long time’ (P2).

Patients valued the pragmatic approach taken by the clinic who did not suggest people stop drinking rather break recovery down into small achievable goals. One patient explained:

‘He made it very clear that he didn’t believe that me going tee-total was the right thing. We discussed that I needed to improve my relationship with alcohol. But he made it very clear to me, he took away, well, the fear, if you like’ (P2).

RAS sessions employed a holistic approach to co-occurring excessive alcohol use and mental ill-health that recognised the impact on all areas of patients’ lives, one patient commenting: *‘We covered everything, my state of mind, my girlfriend, my drinking, what I can do to improve myself, what I can do to stop the drinking’ (P1).* The RAS provider facilitated discussing what had been going on for patients before and after a self-harm episode, to help them develop insight:

‘Was there an increase in self-harm? What was the trigger for that? And allowing the individual to understand their emotions a lot better, and what potentially triggered them … we’re always talking about what they feel, what they want, and how they can achieve that’ (C3).

RAS patients described learning techniques to manage responses to potential risk events (‘*giving me situations and scenarios, so I don’t react quickly’:* P4) and coping strategies to manage alcohol use and mental health, including keeping an alcohol diary, activity schedule, and goal setting. Participants accounts suggested the sessions helped build social and recovery capital, a clinician explained:

‘Because if nothing changes in that context, what is going to keep that individual from relapsing? Because we want to build up on that for the individual, to try to stop them from relapsing, and lay some foundations’ (C3).

Patients described being encouraged to pick-up past hobbies and interests like reading and art and craft possibly neglected while drinking:

‘I used to be a long-distance runner, a lot of years ago. [RAS nurse] told me to start focusing on that, if I could, and I did. I’ve started to keep myself fit, take my mind off the drinking, take my mind off negative thoughts’ (P1)

#### 3.2.3 Theme 3. What could be improved: opportunities and challenges to sustaining and scaling-up the RAS

The RAS clinic provided short-term support, and whilst patients were positive about care received, several would have liked more sessions, one patient explaining:

‘I just had three hours. I think I probably could’ve done with another three sessions because we’d just got to a point where we’d actually … it’s almost like if you imagine you’ve got a jumper, and you start to unpick the hem on the sleeve. I feel like I’d just started to unravel the sleeve when the sessions stopped’ (P10).

Clinicians recognised the shortcomings of the time-limited-support offer, but this allowed them to address immediate safety concerns: ‘…*we can’t fix everything, we can’t solve everything in such a short period of time, but if an individual has presented in services in a mental health crisis, we are able to support them through that period quite safely and effectively’* (C3). Participants also highlighted the limited capacity of the RAS, being delivered by a single clinician it had to be paused during periods of absence:

‘If [RAS nurse’s] off we have nobody else to run that clinic. From an operational perspective that’s difficult and [RAS nurse’s] very aware of that … it’s really unfortunate that we have nobody else to run it’ (C1).

It was clear that any new clinicians would need to combine a positive relational and holistic approach, with awareness of underlying mechanisms driving excessive drinking:

‘Mental health knowledge and experience, as well, comes into play with that, to try and look at the interventions from a life planning, goal setting point of view. What are they doing within their life to change these issues that are ongoing? What’s their motivation? What’s their goal? And having that out with the service user, creating that conversation, and motivational interviewing, really, that identify what their specific treatment requirement is’ (C3).

A patient expanded highlighting that clinicians would require a pragmatic approach:

‘Not feeling embarrassed to write down “I’ve had a drink today”. Because at the last one [service] you weren’t supposed to drink at all and you couldn’t talk about alcohol in that way. You couldn’t say, “I’ve had a drink”. This was different, it allowed you to speak about it without feeling guilty. And if you’d had a drink, to work through why you had a drink’(P9).

Participants stressed the need for a full-time dedicated clinician, without management responsibilities, and graded at an appropriate level of seniority. Clinicians described challenges to service delivery included limited awareness of the RAS’s specific role and place within local care infrastructures, and that some colleagues were unclear about the referral criteria, potentially causing delays in care provision. To address this, clinicians suggested a designated contact be strategically embedded within the care pathway:

‘Educating the access services, educating the psychiatric liaison teams, educating the crisis teams, in every locality, in having that consistent pathway there … being able to explain that to services, I feel, really links the gap nicely. Because generic services, I suppose, you put a referral in, you discuss the referral, and you come up with an opinion or an assumption. However, having that additional person there to advocate for the service user really supports them’ (C3).

The existing RAS team were preparing to set up a new clinic in another location and reflected on other requirements, including integration with existing health services:

‘It would be linking with other services around us to identify what the need is, and getting a clinic sorted by identifying the room, identifying the population you want to work with, and the inclusion criteria, look at the exclusion criteria, and then get the ball rolling. But I suppose that would be linking in with different psychiatric liaison services, and looking at what they’ve used historically, and currently … more services, such as, like, blood borne virus screening, making sure that people’s physical health needs are being adhered to, as well as their mental health needs’ (C3).

Clinicians described practical ‘lessons learned’ from setting up the RAS that could inform scale-up to other locations including developing session content, guidance for measuring core outcomes, and protocols that would need to be established for e.g. referral criteria, non-attenders, lone working, and record-keeping.

Quantitative and qualitative data on co-occurring mental health conditions and quality of life are presented side-by-side in **Tables 3** and **4** and compared in the discussion.

**Table 3:** Co-occurring mental health conditions identified in the quantitative health data alongside patient quotes.

Mental health conditions			Patient experiences
n = 68	n	%	n = 11
Personality disorder	6	8.8	*‘I was going through a really difficult time with my mental health obviously alcohol involved and that makes things a thousand times worse so I was ending up in the [Hospital] constantly, overdoses, self-harm just out of control mental health’.* *‘I’d be told your medication's not working because you’re drinking … I know that alcohol impacts on mental health but like I say I’ve got an addiction with alcohol … I felt like I was being told, this is just my interpretation not saying that’s how it was…“well you go teetotal or we won’t help you”’.*
PTSD	3	4.4	*‘He just told me to take time out and relax and not to think of bad things … to get some of the PTSD problems out of my head’*
Anxiety	14	20.6	*‘I was doing 70 hours a week … I was just absolutely burnt out. I don’t even think it’s a medical term but I actually think I had a nervous breakdown’.* ‘*I also had the added stress of being made redundant during COVID … as a taxpayer and somebody who has always worked, there was very little help available. It took me seven months to find a new job, which was very, very, stressful.’*
Suicidality Depression	1 19	1.5 27.9	*‘I had a suicidal episode’* *‘I was suffering with my mental health because I lost my wife’* *‘I had taken an overdose, but due to being really drunk and having alcohol problems’.* *‘I’d broken up with my wife, I’d pretty much lost everything … and I’ll admit, I mean, I hit the alcohol pretty hard’.* *‘I started drinking when I got cheated on the first time by my boyfriend, and then it just really went downhill from there, and then it was making me really angry’*

PTSD, post-traumatic stress disorder.

**Table 4:** Changes in quality-of-life scores from baseline to follow-up and illustrative patient quotes describing coping strategies found helpful.

Mean ReQoL-20		Quotes
Baseline	Follow-up	
30.8 (range 14 - 53)	46.6 (range 23 – 69)	‘*It was a big deal … for me and done a 360 after’* *‘Reacting to situations. I don’t do that anymore because I feel like that’s what caused most of my problems, just the way I acted, or if I had something in my mind I would want to act on it. Now I just try and think to myself, “That’s not the best thing to do.” I just try and cool myself down’.* *‘[RAS nurse] was really good about that, putting my thoughts into my head where, “Yeah, I don’t need to be depressed. I don’t need to be down, just focus on myself”’.* *‘I’ve still got that where it’s not a guessing game like thinking when did I go to that party when did I have a drink cos obviously with mental health and alcohol my memory is really not good … so I do get quite confused on days and times so having the calendar has been really helpful and nobody has ever suggested that to me before.’* *‘The tools he has given me to use are much better than wherever else I’ve been before. I can use them and they are helping, I feel like I am getting better’.* *‘I know what it’s all about, it’s all about the triggers that cause drinking’* *‘I'm doing well … I don’t think about picking a drink up in any situation. If my life went bad, I would just want a drink to forget about it, but I don’t do that anymore’* *‘I’ve got my blinkers on now and I’ll get up every day, even if it means I do one thing that’s productive every single day then that’s what I’m going to do’.* *‘I’m a more positive person now. I don’t look back on those sorts of problems anymore’.* *‘It’s mind-blowing, like. Because even when I was leaving, like, I was walking out, I don’t know, I was just high on life, if you know what I mean, like I was just like, “Wow, that was amazing.” It’s like, you know, I just released some steam’.* *‘I started picking up books again, because I’m an avid reader’.*

ReQoL-20 scores of 0–49 are within the clinical range; scores of ≥50 indicate the general population range.

## 4 Discussion

Despite the well-evidenced relationship between alcohol use and self-harm, and poor outcomes experienced by many people experiencing these concurrently, research shows persistent care gaps for people affected in the UK and internationally (13, 17, 22–24). We evaluated a new rapid access personalised face-to-face service in North East England for patients with excessive alcohol use, who self-harm, many with co-occurring mental health conditions but deemed unsuitable for specialist alcohol treatment services. Triangulating lived experience of the service with quantitative patient data, we found evidence of positive experiences of care, and health improvements in patients accessing the RAS. Patients described how the intervention enabled them to acquire new coping strategies to better manage their alcohol use and self-harm, and this was further evidenced by improved quality of life scores.

Our findings reflect and build on those of other studies, highlighting the need for tailored support to address the complex overlapping psycho-social factors impacting the behaviour of patients who self-harm and drink alcohol excessively (17, 25, 44, 45). Our data highlight the clinical complexity of this population; many patients accessing the RAS clinic had a co-occurring mental health condition (**Tables 1**, **3**) and were experiencing adverse life events (e.g. relationship breakdown, housing, family and financial concerns, unemployment, bereavement). To address patients’ needs, sessions utilised different therapeutic techniques (e.g. motivational interviewing and psychoeducation) and advice on resuming interests e.g. running. This aligns with research showing the relationship between physical activity, alcohol consumption and distress (46). Other research also found motivational interviewing, cognitive behavioural and psychoeducational approaches helpful for people with excessive alcohol use including those with co-occurring mental ill-health (47–49) though research is required into effectiveness of digital interventions for this group (50).

Patients reported the holistic personalised approach contrasted with previous difficulties accessing care. Despite the ‘no wrong door’ policy for individuals with multiple co-occurring conditions (51, 52), healthcare still focusses primarily on identifying and managing single diagnoses (53, 54). Narrow eligibility criteria can mean individuals face barriers to accessing appropriate mental health care, despite guidance recommending access to preventative psychological interventions (55). Participants in our study described experiencing being ‘blocked’ from or ineligible for mental health support services unless they stopped drinking alcohol. Research with people experiencing excessive alcohol use and depression in North East England also found many encountered a lack of awareness/acknowledgment among providers, of the relationship between alcohol and mental ill-health, and the adverse impact of socio-economic factors on capacity to access support and recovery (22).

Our study demonstrates the potential contribution the RAS, designed for people who drink alcohol excessively but are not dependent, could make to existing healthcare provision. Stakeholders have called for a UK national alcohol strategy and development of secondary preventative interventions for clinical populations which are currently lacking (56, 57). Whilst the patients we spoke with would not meet existing criteria for specialist alcohol services, their alcohol use and mental ill-health were clearly a significant cause of risk and negatively impacted their day-to-day lives and quality of life.

Study strengths include combining quantitative and qualitative data to help understand how the RAS service supports people with excessive alcohol use who present to emergency services following an episode of self-harm; this mixed methods approach facilitates triangulating insights from different perspectives (38, 58). Also focussing on a service innovation which responds to an unmet care need identified in the UK and internationally (11, 13). Focussing on one geographical area could be construed as a limitation, however we gathered data exploring what would be required for transferability to other settings.

This preventative response to alcohol use disorder and mental health aligns with the NHS Long Term Plan’s aim to support people to address harmful lifestyle behaviours, identify and treat avoidable illness early, and exploiting opportunities for intervention during hospital admissions (59). Evidence shows the group targeted by the RAS service are likely to have repeat attendances at the emergency department (60), and in the context of rising numbers of alcohol-related deaths, particularly in North East England (61), the RAS service provides a promising approach for these patients. Our evaluation suggests key ingredients of the service potentially include rapid access to support, personalised holistic sessions considering mental health and alcohol use in parallel and delivered by non-judgemental clinicians. The findings suggest it would be important to explore opportunities to scale-out implementation into other clinical settings; for example, these could include alcohol nurses delivering the service in primary care with appropriate support. This is an innovative service, albeit based on delivery by one therapist. Future research should consider a wider staff base to ensure positive outcomes can be replicated and are not linked to the skills of individual clinicians.

A study strength was completing exploratory analysis using a standardised quality of life measure (ReQOL-20). However, scores at both baseline and follow-up were only available for fifteen people and this potentially introduces bias. It may be that those people who completed treatment sessions were more positive about the service compared with those people who disengaged early, and our findings would not capture or reflect the perspectives of those people in the latter group. We completed exploratory statistical significance testing; however, this is a small-scale study and there was no control group. Furthermore, patients in the interviews were invited to participate by the RAS clinician and we cannot assume the views of the eleven people interviewed would be the same as RAS patients who did not participate in the study. Moreover, while participant feedback was positive, the sample was again relatively small. We did not have data on the proportion of the patients accessing RAS who received concurrent aftercare from other sources e.g. the crisis team, or on the numbers of patients who would previously have been referred directly to the crisis team but were instead referred to the RAS and this is an area for future research. Future research is needed regarding implementation of similar clinics and any barriers in the context of changes to UK NHS mental health commissioning and the role of UK local integrated care boards who are tasked with commissioning and planning services for regional populations (59, 62, 63).

## 5 Conclusion

The findings contribute to an acute evidence gap in appropriate models of care for patients with co-occurring excessive alcohol use, self-harm and poor mental health. The data shed light on what was valued by patients and clinicians and how the approach could be implemented in other settings. The findings have potential to inform future commissioning decisions, innovation and service development in mental health and other clinical settings in the UK and internationally.

## Data Availability

The datasets generated and/or analysed during the current study are not publicly available due to confidentiality but are available from the corresponding author on reasonable request. Requests to access the datasets should be directed to amy.odonnell@ncl.ac.uk.
