# Supplementary material for: Personalised care for people with excessive alcohol use following an episode of self-harm: a mixed methods community case study in a psychiatric liaison team
Source: Front Psychiatry. 2025 Aug 22;16:1608804. doi: 10.3389/fpsyt.2025.1608804 (PMC12411859; doi:10.3389/fpsyt.2025.1608804)
Supplement: Supplementary file 1 [file DataSheet1.pdf]

Online Supplementary Materials 1 (OS1)

SERVICE EVALUATION

PATIENT INTERVIEW TOPIC GUIDE

INTRODUCTION

- Thank you for taking the time to meet and share your experiences and views about the clinic (i.e. the sessions you attended with XX)

- Anything you share here today will be treated with confidence. The names of any places or people you mention will be changed to ensure you can't be identified. However, if you tell me something which makes me concerned about the safety of yourself or other people, I will need to tell someone about this. I would tell you who I am going to speak to and what I am going to tell them.

- Can I check that you have read the study participant information sheet, do you have any questions for me?

- Work through written consent or reaffirm consent if consent forms already signed.

- If there are any questions you don't want to answer that is absolutely fine. If you want to stop at any time that is fine too. There are no right or wrong answers the interview is about your experiences.

- Are you ok for me to turn the recorder on and start the interview?

BACKGROUND

First, we would like to find out a little about you and your current work/living situation:

Please could you start by telling me a bit about your everyday life at the moment? *(Probe: living circumstances, social/familial support, paid employment, children/caring responsibilities)*

As you know, the aim of this evaluation is to find out what has worked well for people accessing the clinic and what might help improve the service. We are keen to understand if the clinic might offer a better approach to supporting people who struggle with heavy drinking and mental health issues that could be introduced more widely across the region.

Please could you tell me about your own experience of drinking and/or mental ill-health including self-harm? *(Probe: how long they have been experiencing these, how they feel /think the two relate, how it affects daily life)*

Please could you tell me which health and social care services / groups you have previously sought or received help from about your drinking and/or mental health? *(Probe: try to get a sense of the trajectory, whether used multiple services, referrals from one to another, sharing information/data, referrals from one to another, whether mental health has caused barriers to accessing support)*

ACCESSING SUPPORT THROUGH THE CLINIC

Next, we would like to find out about your experience of accessing support through the clinic (sessions with the nurse). We would like to hear about your journey into and out of the service, what you felt worked well and any aspects that could be improved.

Before you attended the sessions

Can you tell me about the self-harm episode that led to you being referred to the clinic? *(Probe: what was happening in their lives at that point, what led them to seek help, did anyone encourage them to seek help, why did they go to the ED, did they try any other service before that?)*

Can you tell me how you were made aware of the clinic, and what information you were given at the time? *(Probe: who told you, what did they tell you, what information were you given)*

Can you tell me about the process of getting to the clinic? For example, what made you want to attend? How soon after the self-harm episode did you attend the clinic? What were you feeling like at the time? How did you get to the appointment?

The sessions themselves and afterwards

What did you discuss and do during the sessions you had with the nurse?

What aspects of the sessions did you find most helpful at the time; what aspects of the sessions have you found most helpful since the sessions finished?

Did the nurse tell you about any other services or support? If yes, what were these, and have you gone on to access any services that they told you about? *(probe, about what support these services have provided) (If they were told about services / support but haven't access ask about reasons for this)*

What if anything has changed about your drinking and/or mental ill-health since you finished the sessions at the clinic? *(probe: to what extent they feel this relates to involvement with the clinic)*

What if anything didn't you like about the clinic?

What do you think could be improved about the clinic?

CLOSING QUESTIONS

Is there anything else you would like to say about your experience of the topic of this interview? Or about anything else we have discussed today?

END OF INTERVIEW

- Thank participant for their time and switch off recorder.
- Complete demographic form
- Discuss how they feel after the interview, ensure they have a copy of the study Participant Information Sheet. Check address to send voucher.

Online Supplementary Materials 2 (OS2)

SERVICE EVALUATION

STAFF INTERVIEW TOPIC GUIDE

INTRODUCTION

- Thank you for taking the time to meet and share your experiences and views about the clinic
- Anything you share here today will be treated with confidence. The names of any places or people you mention will be changed to ensure you can't be identified.
- Can I check that you have read the study participant information sheet, do you have any questions for me?
- Work through written consent.
- There are no right or wrong answers the interview is about your views and experiences.
- Are you ok for me to turn the recorder on and start the interview?

BACKGROUND

First, we would like to find out about the work that your organisation does and your personal role and responsibilities.

Could you briefly tell me about your background and experience, and the role you hold at this organisation?

Could you tell me about any services you / your organisation provide for people who are experiencing co-occurring depression and heavy drinking? *(Probe: focus of services; whether mostly depression or alcohol etc; how the service defines depression and heavy drinking, geographical/administrative remit)*

THE CLINIC

Next, we want to hear about your involvement with the clinic

Could you tell me about how you are involved with the clinic, and how it fits with your wider role and patient population you work with?

What is your understanding of why the clinic was established, and any gaps in care it is intended to address?

Could you tell me about the process of setting up the service, and what factors have needed to be considered in this process?

Please could you tell me about the focus and content of the sessions, and your understanding of why they are delivered in this way?

What do you personally think is working well about the service, and what is enabling this? *(Probe: for patients, staff)*

What do you perceive to be the key outcomes of the clinic so far?

What could be better about the service? What would need to be done to make these happen?

POTENTIAL FOR SCALE-UP

Now we would like to understand the extent to which the service could be scaled up and what would need to be in place for this to happen:

Do you feel the clinic has the potential to be scaled up to other sites or NHS trusts? *(Probes: If no, probe why not? If yes, probe why?)*

What key factors would need to be considered for the project to be scaled up?

What potential do you feel it has to improve care if is scaled up? *(Probe: which patient populations and gaps in care would it be filling)*

CLOSING QUESTIONS

Do you have any other thoughts or suggestions on the clinic, or anything about the topic of the interview?

END OF INTERVIEW

Thank participant for their time and switch off recorder.

Online Supplementary Materials 3 (OS3)

SERVICE EVALUATION

PATIENT INTERVIEW DEMOGRAPHIC FORM

This form will take approximately five minutes to complete. It will help the evaluation team to compare the views and experiences of patients taking part in the evaluation based on factors such as the area where they live or their age. For example, we might compare the interview data of people who have high depression scores and those who have lower depression scores. The information will be kept anonymously and confidentially and will only be accessible to the evaluation team.

Q1. What is your month and year of birth? ☐☐☐☐☐☐

Q2. What is your home postcode? ☐☐☐☐☐☐☐

Q3. What is your gender? Please tick one box only

- ❍ Male
- ❍ Female
- ❍ Other, *please specify*________________________________
- ❍ Prefer not to say

Q4. What is your ethnic group? Please tick one box only

- ❍ White
- ❍ Mixed/Multiple ethnic groups
- ❍ Asian/Asian British
- ❍ Black/ African/Caribbean/Black British
- ❍ Other ethnic group, *please specify*________________________________
- ❍ Prefer not to say

Q5. What is the highest level of education that you reached? Please tick one box only

- ❍ Left school before completing GCSEs/ O Levels
- ❍ GCSEs/O Levels
- ❍ A Levels/Scottish Highers or International Baccalaureate
- ❍ Apprenticeship
- ❍ Professional qualifications
- ❍ First Degree
- ❍ Higher degree or above

❍ Other qualification, *please specify*______________________________

❍ Prefer not to say

Q6. What best describes your current status? Please tick one box only

❍ Married or living with your partner

❍ Single

❍ Divorced

❍ Have a partner but not living together

❍ Other, p*lease specify*______________________________

❍ Prefer not to say

Q7. What best describes your current employment? Please tick one box only

❍ In paid Full-time employment

❍ In paid Part time employment

❍ Self-employed or freelance

❍ Studying or in training

❍ On Maternity Leave or Sick Leave from full time employment

❍ On Maternity Leave or Sick Leave from part-time employment

❍ Not in paid employment
